# Screening Utility of Ambient Pressure Tympanometry for the Detection of Radiologic Correlates of Subjective Pulsatile Tinnitus

**DOI:** 10.1097/ONO.0000000000000097

**Published:** 2026-09-07

**Authors:** Christian Bernard, Cyrus Khorassani, Monica Amarante, Greta Stamper, Joseph Breen, Mallory Raymond

**Affiliations:** 1 Department of Otolaryngology—Head and Neck Surgery, Mayo Clinic Florida, Jacksonville, FL; 2 Department of Otolaryngology—Head and Neck Surgery, Geisinger Medical Center, Danville, PA.

**Keywords:** pulsatile tinnitus, ambient pressure tympanometry, imaging

## Abstract

**Objective:**

To determine the sensitivity and specificity of ambient pressure tympanometry (APT) for the detection of a radiologic correlate of subjective pulsatile tinnitus (PT)

**Study Design:**

Retrospective review.

**Setting:**

Tertiary care clinic.

**Patients:**

107 adults (147 ears) with PT who underwent APT between 2021 and 2025.

**Interventions:**

Blinded audiologists classified APT tracings as pulse-synchronous or not.

**Main Outcome Measures:**

Sensitivity and specificity of APT using any radiologic diagnosis or surgeon-defined diagnosis as 2 separate gold standards.

**Results:**

Of 137 (93.2%) ears that underwent imaging, 63 (46%) had a pulse-synchronous APT pattern. Fifty-one (37.2%) had a radiologic correlate, and 65 (44.2%) were given a surgeon diagnosis. Respectively, APT sensitivity, specificity, positive predictive value (PPV), and negative predictive value (NPV) were 51%, 57%, 41.3%, and 66.2% for a radiologic correlate and were 43.1%, 54.9%, 43.1%, and 54.9% for a surgeon diagnosis. In a subgroup of 86 patients (116 ears) with a chief complaint of PT, APT sensitivity, specificity, PPV, and NPV for a radiologic correlate were 56.9%, 54.2%, 46%, and 55.2%, and for a surgeon diagnosis were 43.6%, 54.1%, 46.2%, and 51.6%.

**Conclusion:**

With a low sensitivity and specificity, APT may not be a reliable screening tool for the detection of a radiologic correlate of subjective PT. Clinicians should continue to pursue radiologic evaluation of PT despite a negative APT and counsel patients on the limits of imaging and the potential need for multiple modalities for the detection of subjective PT sources.

Pulsatile tinnitus (PT) refers to the auditory symptom of perceiving repetitive rhythmic sound without a known stimulus (1,2), but for which an underlying cause may be found in up to 70% of patients (3). Etiologies generally include venous and arterial abnormalities such as carotid artery stenosis, arteriovenous malformations, and sigmoid sinus wall abnormalities (SSWAs), as well as structural defects such as superior semicircular canal dehiscence (SSCD) and encephaloceles, and mass lesions of the temporal bone or lateral skull base (4,5).

The initial evaluation of PT begins with a focused history and complete neurotologic exam to identify obvious pathology and evaluate for objective PT using auscultative maneuvers (3). However, these are often nonspecific or noncontributory, leading clinicians to proceed to imaging (4). The choice of initial imaging is aimed at identifying the most likely or threatening sources of PT while minimizing cost and invasiveness (6,7). Previously suggested algorithms recommend that initial imaging (magnetic resonance imaging [MRI], ultrasound, computed tomography [CT] of the temporal bone, or CT angiography) be guided by clinical assessment and suspected etiology, with additional modalities obtained as needed (3). Despite a clinically well-justified stepwise approach, patients often undergo multiple imaging studies without reaching a definitive diagnosis (3,6), while others may be forced to undergo an inadequate workup due to the inability to obtain various imaging modalities in some settings. Thus, there is a need for a simple, office-based screening tool that could risk stratify patients with PT to better inform the need for and type of imaging.

APT, a 15–20 second audiological examination performed using a microphone to record changes in ear canal volume and thereby tympanic membrane movements, has been studied as a potential screening tool. APT was found to have a 66.7% sensitivity and 72.1% specificity in patients with SSCD (8) and a 53.3% sensitivity and 93.9% specificity in patients with patulous Eustachian tube (PET) dysfunction (9). However, there has otherwise been very limited data (10) to date on the potential of APT as a screening tool for the detection of any radiologic correlate of subjective PT. Therefore, the objective of this study was to evaluate the utility of APT as a screening tool for the identification of any radiologic correlate of subjective PT.

## METHODS

### Patient Identification

This retrospective study was approved by the Institutional Review Board (IRB-22-013130). The institution’s patient data explorer (PDE), a self-service data exploration and retrieval tool of structured and unstructured data, was searched for patients for whom a complaint of “pulsatile” was documented in the electronic medical record (EMR) between December 1, 2021 and May 2, 2025 by a neurotologist. The initial data search identified 537 unique patients. Patients were then screened for a positive PT complaint, as opposed to a denial, using the question, “Did the patient report pulsatile tinnitus?” Using the PDE integrated large language model (LLM), ACCRUE-L (Automating the Creation of Clinical Registries Using Existing LLMs), 178 patients denied PT, leaving 359 patients to be screened further. These patients were further screened manually for the following inclusion criteria: adults (≥18 years of age) who complained of PT, had complete audiometric testing including pure tone audiometry, tympanometry, and APT, as well as types A, Ad, or As tympanograms, and an average air-bone gap (ABG) of less than 25 dB (calculated by the difference in 4-frequency [500, 1000, 2000, 4000 Hz] air and bone pure tone averages) in the symptomatic ear. Patients were excluded if they were less than 18 years of age, had a history of otologic surgery (excluding pediatric tympanostomy tubes), otologic trauma, or Meniere’s disease, or had type B or C tympanograms. After screening for inclusion criteria, 107 patients (147 ears) were identified.

International Classification of Diseases-10 diagnosis codes for PT were not used to identify the cohort or as an inclusion criterion because these codes were not uniformly applied by each neurotologist and would likely have biased the cohort toward patients in whom a definitive source had been identified. However, to study a population with a more specific complaint of PT than that captured in the methodology described above, a subgroup analysis of patients with a primary complaint of PT, defined as either a chief complaint of PT or a diagnosis of PT included in the assessment and plan of the clinical visit note, was performed. PT that was mentioned only incidentally or grouped among other more highly prioritized symptoms without being addressed in the clinical assessment was considered a secondary complaint. This stratification yielded a primary complaint subgroup of 86 patients (116 ears).

### Data Collection

The EMRs of the 107 patients who met the inclusion criteria were reviewed. Data were recorded in an internal REDCap survey. Data collection included patient demographics, comorbidities relevant to PT, PT laterality, and tympanogram classification. Additionally, data regarding the performance of imaging tests and their associated interpretations were recorded, both for imaging performed within and outside the institution. Institutional imaging workup was guided by symptom description, physical examination, and review of any outside studies. For patients with a venous pattern PT and without an obvious source of PT on examination (eg, middle ear pathology), institutional practice is to obtain either a noncontrast or venous-phase contrast photon-counting CT temporal bone, if not already completed. Likewise, if not already performed, arterial-focused or structurally focused imaging, including both an MRI and magnetic resonance angiography (MRA), was pursued if the clinical presentation suggested an arterial or unclear pattern. Finally, if additional symptoms or previous imaging raised concern for idiopathic intracranial hypertension (IIH), general institutional practice includes referral to neuro-ophthalmology and a cerebrospinal fluid dynamics neurologist for intraocular pressure assessment and lumbar puncture. Additional imaging was generally guided by the findings, or lack thereof, of the initial images. Overall, imaging options included CT of the temporal bone without contrast, CT of the head without contrast, CT venogram (CTV) of the head or the temporal bone, CT angiogram (CTA) of the head or temporal bone, CTA of the neck, MRI of the head/brain, MR venogram of the head/brain, MRA of the head/brain, MRI of the neck, and MRA of the neck. Radiologist interpretations were recorded and documented as either supporting or not supporting a source of PT. Finally, surgeon diagnoses were separately included in data collection as the possibility of disagreement between radiologist and surgeon radiologic interpretation was anticipated. Surgeon diagnoses, neither blinded to APT nor radiology findings, were extracted from the assessment or diagnoses sections of the consultation or final progress note addressing the source of the PT.

### Ambient Pressure Tympanometry

Per institutional audiology protocol, APT is performed for any patient who reports pulsatile tinnitus during their clinical evaluation. APT tracings were obtained by trained audiologists and audiology residents in sound-treated booths. Patients were seated upright in a chair and instructed to remain still and quiet while breathing normally as APT tracings were obtained. APT testing was performed using the Interacoustics Titan Middle Ear Analyzer (Middelfart, Denmark) controlled by the Titan Suite software on the nearby computer. The Titan probe was inserted appropriately into the ipsilateral ear canal of the patient. The audiologist/audiology resident ensured an adequate seal was acquired before initiating testing. Next, a 226 Hz probe tone at 85 dB SPL was presented to the ear canal for 20 seconds without the introduction of air pressure, and tympanic membrane movement was recorded. These recordings were uploaded to the EMR as single images.

**FIG. 1.**
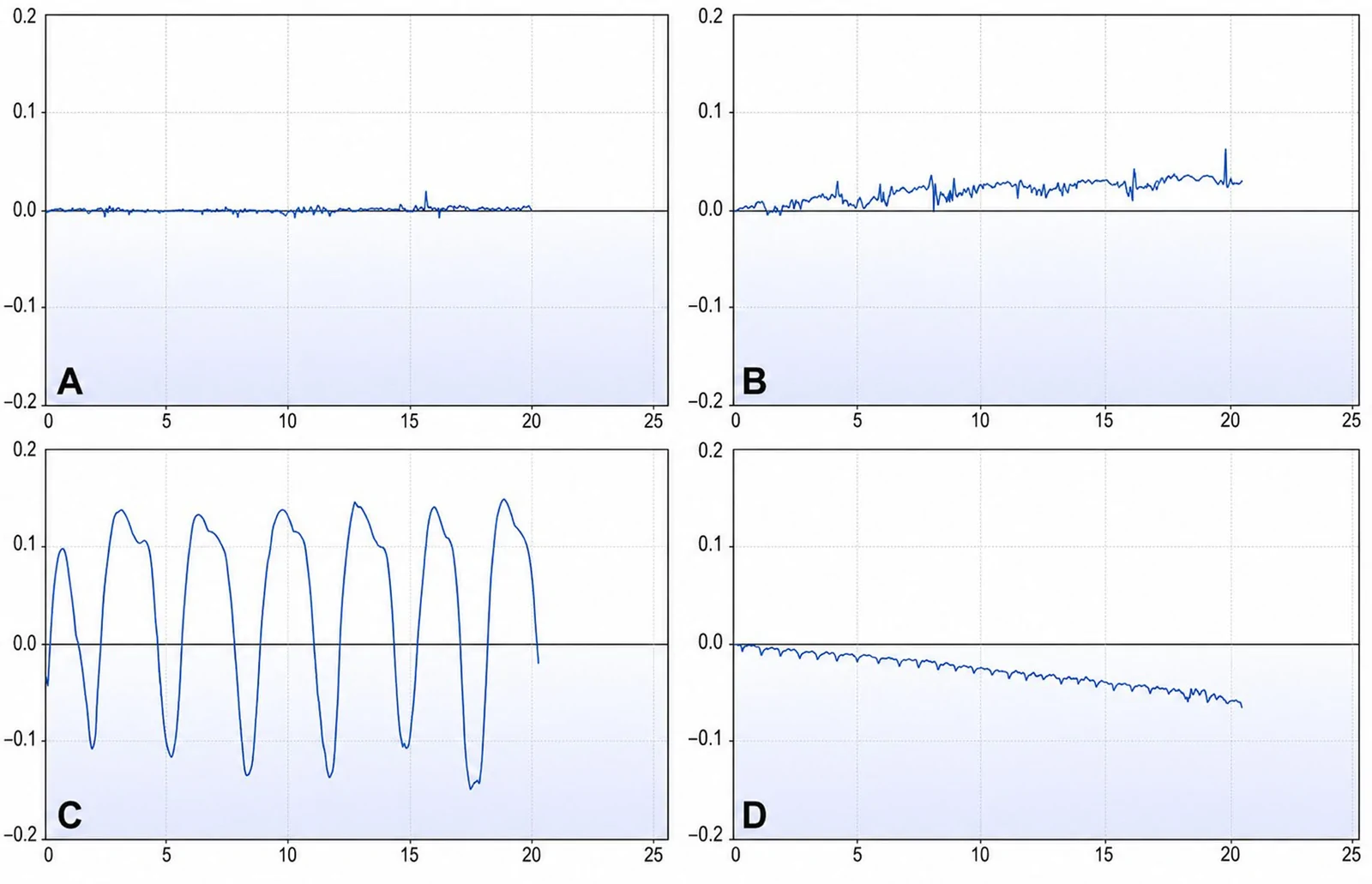
Examples of APT tracings with no fluctuations (A), noisy fluctuations (B), respiratory-synchronous waveform patterns (C), and pulse-synchronous waveform patterns (D). The X-axis shows the time in seconds (20 seconds per tracing), and the Y-axis shows the volume of the external auditory canal. APT, ambient pressure tympanometry.

For the purposes of this study, APT tracings were isolated from medical records and provided in a PowerPoint deck as single images to 2 audiologists blinded to patient identity and diagnosis. The audiologists independently reviewed the APT tracings and were prompted to classify the APT waveform (9) for each ear based on the following predefined categories: no fluctuation, noisy fluctuation, respiratory-synchronous waveforms (6–30 peaks/min), and pulse-synchronous waveforms (40–100 peaks/min) (Fig. 1). Waveform tracings that met the criteria for pulse-synchronicity over at least 50% of the 20-second tracing continuously were also classified as pulse-synchronous. Finally, waveforms that included respiratory-synchronous waveforms overlaid on pulse-synchronous waveforms were classified as respiratory-synchronous, as it was presumed a priori that any respiratory-synchronous pattern would point to a diagnosis of patulous Eustachian tube dysfunction (ETD). Disagreements were resolved by discussion between the 2 audiologists and the senior author.

### Statistical Analysis

Demographic factors were descriptively analyzed using Excel. APT sensitivity, specificity, positive predictive value (PPV), and negative predictive value (NPV) were calculated using both a positive radiologic correlate and a definitive surgeon diagnosis as the diagnostic gold standards. These calculations were repeated for the primary PT complaint subgroup.

## RESULTS

Of 107 patients with PT who underwent APT, 67 (62.6%) complained of unilateral tinnitus and 40 (37.4%) complained of bilateral tinnitus. The average patient age was 57.3 ± 13.0 years; 90.7% (n = 97) of patients were white, and 77.6% (n = 83) were female (Table 1). Relevant comorbidities were common (Table 1). On standard tympanometry of the 147 ears included in the analysis, 90.5% (n = 133) displayed type A tympanograms, 5.4% (n = 8) type As, and 4.0% (n = 6) type Ad tympanograms. The average ABG was 2.1 ± 3.9 dB, with no patients exceeding 15 dB. APT displayed a pulse-synchronous pattern for 65 (44.2%) ears, a respiratory-synchronous pattern for 8 (5.4%) ears, and a noisy or nonfluctuating pattern for 47 (32%) and 27 (18.4%) of ears, respectively.

**TABLE 1.** Demographic and clinical characteristics of 107 patients with subjective pulsatile tinnitus

Demographics	Value
Mean age (years) ± SD	57.3 ± 13 years
Sex (n, %)	
Female	83, 77.6%
Male	24, 22.4%
Race (n, %)	
White	97, 90.7%
Black	6, 5.6%
Asian	1, 1%
Native American	1, 1%
Not reported	2, 1.9%
Comorbidities (n, %)	
Hypertension	42, 39.3%
Hyperthyroidism	5, 4.7%
Valvular disease	4, 3.7%
Arrhythmia	4. 3.7%
Idiopathic intracranial hypertension	4, 3.7%
Atrial fibrillation	2, 1.9%
Cardiomyopathy	1, 1%
Congestive heart failure	1, 1%
Carotid stenosis	1, 1%
None	56, 52.3%

### Radiologic Workup

**FIG. 2.**
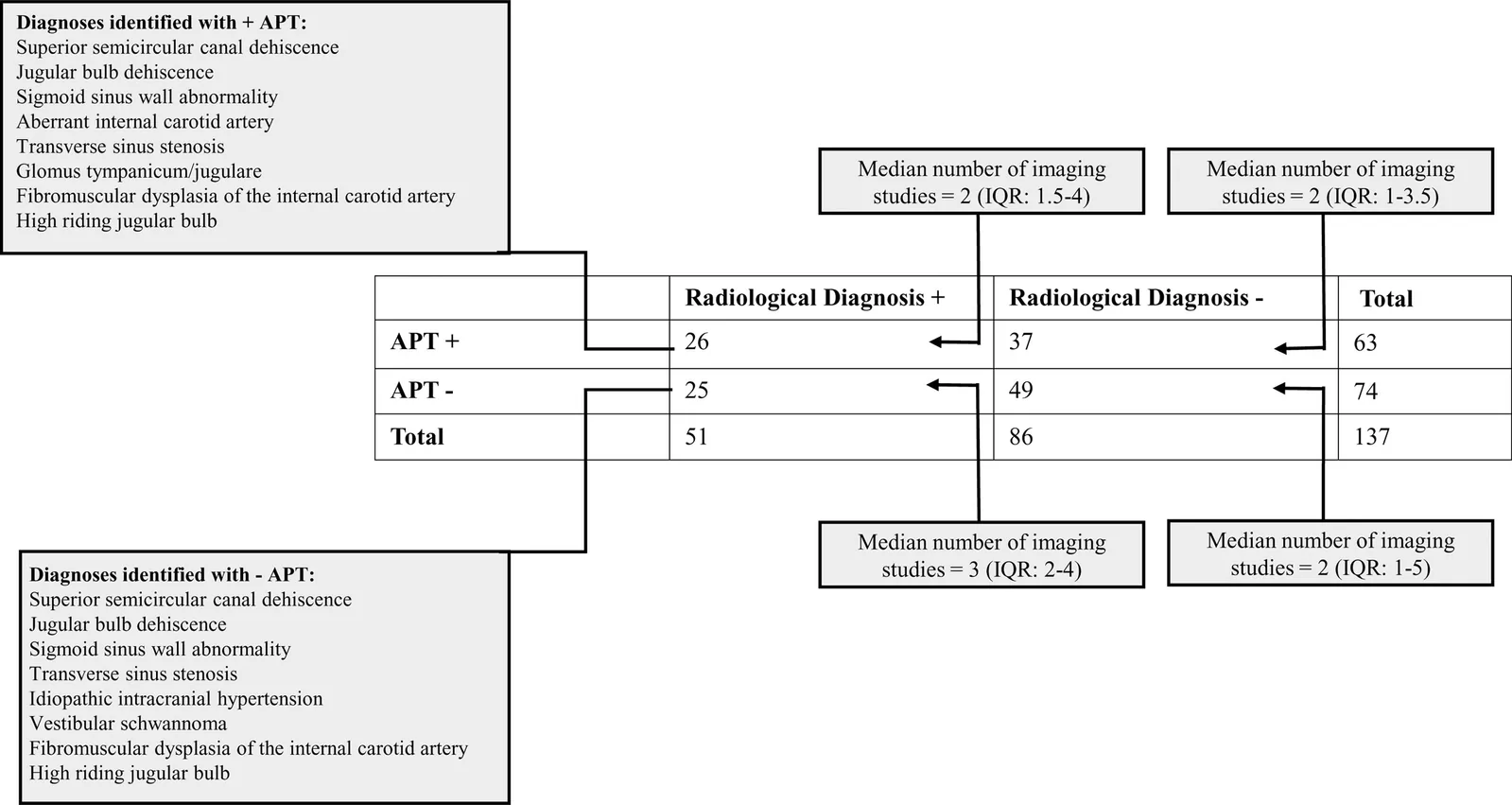
Two-by-two table showing APT results by radiologic findings. APT, ambient pressure tympanometry.

Radiologic testing was performed for 137 (93.2%) ears (Table 2). Radiological diagnoses were identified for 51 (37.2%) ears, with 6 ears having 2 diagnoses. The most common diagnoses included: SSCD (16, 28.1%), SSWAs (8, 14%), high-riding jugular bulb (7, 12.5%), transverse sinus stenosis (TVSS) (6, 10.5%), fibromuscular dysplasia of the carotid artery (7, 12.3%), paraganglioma (4, 7%), and jugular bulb dehiscence (3, 5.3%) (Table 3). The median number of imaging studies performed for patients both with and without an identified radiologic correlate was 2 (IQR: 1–3). Of ears with positive imaging findings, 26 (51%) had pulse-synchronous APT waveforms. APT sensitivity, specificity, PPV, and NPV for any radiologic finding were 51%, 57%, 41.3%, and 66.2%, respectively (Fig. 2). Pulse-synchronous APT findings for ears with positive radiologic interpretations were additionally evaluated separately and were found for 3 (37.5%) with SSWAs, 10 (62.5%) with SSCD, 3 (30%) with high-riding jugular bulbs or dehiscence, 4 (66.7%) with TVSS, 2 (33.3%) with fibromuscular dysplasia, and 4 (100%) with paragangliomas.

**TABLE 2.** Total number of radiological tests performed for 99 patients (137 ears) with subjective pulsatile tinnitus

Type of imaging	Total count	Positive radiologic correlate count (ear level)
CT temporal bone	66	36
MRI head/brain	59	10
CTA head	29	9
CTA neck	28	7
MRV headz/brain	27	9
MRA head/brain	18	2
CT head	15	0
U/S neck	12	0
MRA neck	12	1
CTV head	5	2

CT indicates computed tomography; MRI, magnetic resonance imaging; MRV, MR venogram.

**TABLE 3.** Ear-level diagnoses arrived at by both radiology interpretations alone or by surgeons

Diagnostic sources of subjective PT	Radiology diagnosis (ear level, total = 57)	Surgeon diagnoses (ear-level, total = 69)
Sigmoid sinus wall abnormalities	8, 14%	12, 17.4%
Superior semicircular canal dehiscence	16, 28.1%	10, 14.5%
Patulous Eustachian tube dysfunction	N/A	9, 13.8%
Idiopathic intracranial hypertension	1, 1.7%	6, 8.7%
Middle ear myoclonus	N/A	6, 8.7%
Obstructive Eustachian tube dysfunction	N/A	6, 9.2%
High-riding jugular bulb	7, 12.3%	2, 2.9%
Glomus tympanicum	2, 3.5%	2, 2.9%
Fibromuscular dysplasia	7, 12.3%	2, 2.9%
Sensorineural hearing loss	N/A	2, 2.9%
Jugular bulb dehiscence	2, 3.5%	2, 2.9%
Glomus jugulare	2, 3.5%	2, 2.9%
Vestibular schwannoma	2, 3.5%	2, 2.9%
Carotid stenosis	1, 1.8%	1, 1.4%
Vascular malformation	1, 1.8%	1, 1.4%
Otosclerosis	1, 1.8%	1, 1.4%
Bruxism	N/A	1, 1.4%
Aberrant internal carotid artery	1, 1.8%	1, 1.4%
Bony dehiscence of petrous carotid artery	0	1, 1.4%
Transverse sinus stenosis	6, 10.5%	0

### Surgeon Diagnoses

**FIG. 3.**
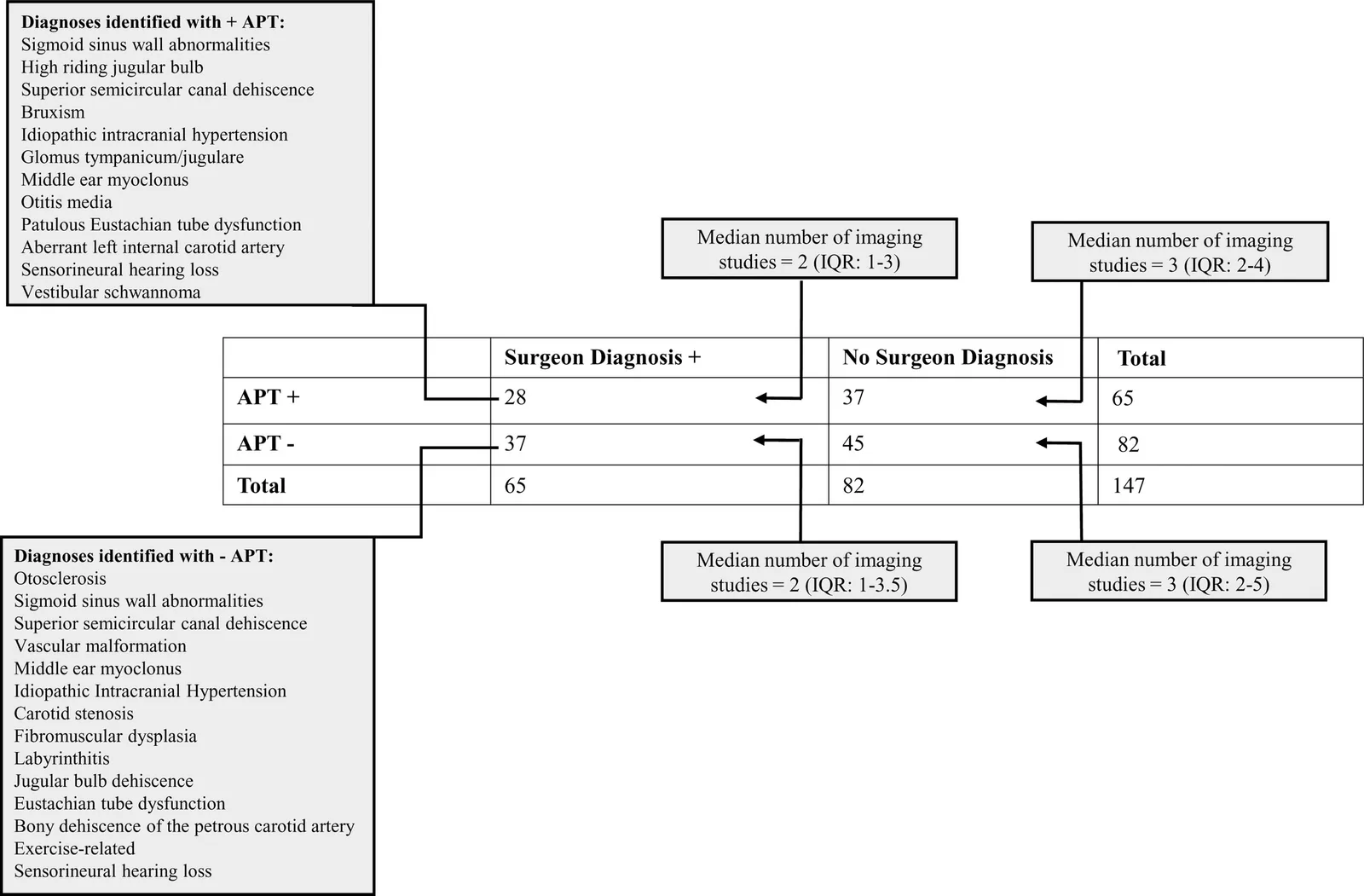
Two-by-two table showing APT results by surgeon-provided diagnoses. APT, ambient pressure tympanometry.

A surgeon diagnosis was provided for 65/147 (44.2%) ears, with 4 ears given 2 diagnoses. Diagnoses included SSWAs (12, 17.4%), sequelae of obstructive ETD (6, 9.2%), patulous ETD (9, 13.8%), SSCD (10, 14.5%), IIH (6, 8.7%), middle ear myoclonus (6, 8.7%), paraganglioma (4, 5.8%), high-riding jugular bulb (2, 2.9%), and fibromuscular dysplasia of the carotid artery (2, 2.9%) (Table 3). Twenty-eight (43.1%) ears with a definitive surgeon diagnosis had a pulse-synchronous APT waveform pattern. APT sensitivity, specificity, PPV, and NPV were 43.1%, 54.9%, 43.1%, and 54.9%, respectively, when using a surgeon diagnosis as the gold standard (Fig. 3). Pulse-synchronous APT waveforms were found for 2 (16.7%) with SWAAs, 4 (44.4%) with patulous ETD, 1 (20%) with sequelae of obstructive ETD, 7 (70%) with SSCD, 4 (66.7%) with IIH, 2 (33.3%) with middle ear myoclonus, 4 (100%) with paraganglioma, and 1 (50%) with a high-riding jugular bulb. Additionally, rare pathologies found to be associated with a pulse-synchronous APT waveform were an aberrant internal carotid artery (1), bruxism (1), and an intracanalicular vestibular schwannoma (1).

### Diagnostic Discrepancies

Of 86 ears without any radiologic correlate provided on imaging interpretation, 23 (26.7%) ears were still provided with a surgeon diagnosis. Of these ears, 17 (73.9%) were diagnosed with sources of their symptoms that could not be identified with imaging, including middle ear myoclonus, PET dysfunction, sensorineural hearing loss, obstructive ETD, and bruxism. Additionally, of the 10 ears for which no imaging was performed, 7 (70%) were still given a surgeon diagnosis; these included middle ear myoclonus, obstructive ETD, and PET dysfunction.

### Primary Complaint Subgroup Analysis

A subgroup analysis was performed on 86 patients (116 ears) with PT classified as their primary complaint. Of 116 ears, 52 (44.8%) had a pulse-synchronous APT waveform. Imaging was available or performed for 108 (93.1%) ears. A radiologic PT correlate was identified for 49 (45.4%) ears. APT sensitivity, specificity, PPV, and NPV for a radiologic correlate were 56.9%, 54.2%, 46%, and 55.2%, respectively. A surgeon diagnosis was provided for 55 (47.4%) ears; APT sensitivity, specificity, PPV, and NPV were 43.6%, 54.1%, 46.2%, and 51.6%, respectively. These values were comparable to the full cohort, suggesting that inclusion of patients with secondary PT complaints did not meaningfully change the APT screening utility.

## DISCUSSION

APT is a cost-effective, accessible, and noninvasive test that can demonstrate rhythmic wave patterns in the evaluation of numerous PT pathologies (11). In this study, we present the only series to date evaluating the utility of APT as a screening tool for the detection of a radiologic correlate of subjective PT across a spectrum of known sources of PT. In this series of 137 imaged ears, APT had a relatively low sensitivity and specificity for the detection of a radiologic source of PT, suggesting that APT may not be a reliable screening tool to guide decision-making to pursue radiologic workup of subjective PT. In other words, in patients with complaints of subjective PT who are found to have negative APT waveforms, a radiologic source of PT may still be detected approximately half of the time. Likewise, in patients found to have pulse-synchronous APT waveforms, approximately half may still ultimately go undiagnosed.

While detection rates of etiologic sources of PT have been reported to be approximately 70% or above (5), in this series, less than half of the studied ears had a radiologic correlate documented by a radiologist or received a definitive surgeon diagnosis. One explanation is the comprehensive nature of patient identification used in this study. By harnessing an LLM, we isolated our cohort by identifying patient-expressed complaints of subjective PT from HPIs, rather than surgeon-diagnosed PT CPT codes, likely generating a more inclusive cohort. This approach was chosen to mimic the reality of clinical practice, which starts with a patient complaint rather than a diagnosis. To address the concern that this approach may have suppressed APT’s utility, a subgroup analysis restricted to patients with PT as a primary complaint yielded comparable results, confirming that the limited screening utility was not an artifact of being overly inclusive.

Disagreement between radiology interpretations and surgeon diagnoses was common, which suggests that radiology testing alone may not be a reliable gold standard for the diagnosis of sources of subjective PT. The most utilized imaging modalities were CT and MRI, aligning with what has been previously recommended (6,7); however, many were performed outside of this tertiary care center and varied in quality of both images acquired and detail of interpretations. This qualitative observation supports a recommendation for evaluating physicians to remain expert in their own radiologic reviews of sources of subjective PT. Highlighting this point, 2 common diagnoses for sources of subjective PT were SSCD and SSWAs, both of which displayed a notable discrepancy between the 2 diagnostic methods. Discrepancies between radiologic SSCD and surgeon-provided diagnoses of SSCD, for example, are likely in part related to the complexity of the presentation and evaluation, in addition to the resolution limits of imaging (12). Still, there was a relatively comparable rate of pulse-synchronous APT patterns found for patients with either a positive radiologic correlate or a surgeon-provided diagnosis of SSCD (8). Additionally, there was a comparable rate of pulse-synchronous patterns found in this cohort as in others, supporting APT’s potentially higher utility in patients with a high pretest probability of being found to have SSCD (8).

Despite gold standard diagnostic discrepancies in the evaluation of SSWA, there were low rates of pulse-synchronous APT patterns for both positive radiologic correlates and surgeon diagnoses. These findings might suggest differences in pathophysiologic mechanisms for the perceptions of pulsations between SSCD and SSWA and their activity against the tympanic membrane, outlined previously (8,10). Interestingly, in previous studies on patients with PT, SSWAs were identified in a larger percentage of patients with PT than were identified in this cohort (13–15). Differences in prevalence or patient populations could thus influence the screening utility of APT. In the study by Sayyid et al, (11) for example, 4/6 ears with SSWAs were found to have a pulse-synchronous APT, representing a higher rate than identified here. As noted by that group, positive APT tracings for these patients could also originate in part from co-existing pathology such as IIH, high-riding jugular bulb, jugular bulb dehiscence, TVSS, and SSCD; thus again pointing to the limitations of radiology as a gold diagnostic standard.

While this study was conceived for the purpose of evaluating the clinical utility of APT for both comprehensive otolaryngologic and neurotologic clinicians, if we assume that the neurotologist’s diagnosis represents the gold standard diagnostic “test” for this cohort, then some notable inconsistencies should be pointed out. First, there was a relatively high rate of pulse-synchronous APT patterns among IIH diagnoses. One of these patients had additional SSWA; 1, with bilateral complaints and bilateral pulse-synchronous APT patterns, had imaging that had not revealed any SSWAs or TVSS, though a lumbar puncture before her visit had confirmed IIH; and one was suspected to have IIH with an additional underlying concern for SSWA and SSCD, and was initiated on a trial of Diamox, ultimately being lost to follow-up. Thus, it is difficult to hypothesize on the relationship between pulse-synchronous APT findings and IIH, SSWA, and SSCD. Second, we would not have hypothesized that any patients diagnosed with middle ear myoclonus would have a pulse-synchronous APT pattern. One patient described both a pulse-synchronous and rapid clicking tinnitus (which was the more bothersome complaint) and had already undergone an unrevealing CTA of the head and MRI of the brain. The other had similar complaints and subsequently underwent an MRI of the brain, which was unrevealing. Interestingly, this patient also had bilateral pulse-synchronous patterns. We have observed a number of additional patients in clinical practice with unilateral complaints but pulse-synchronous APT patterns bilaterally; however, owing to the focus on the evaluation of subjective complaints, we did not analyze waveforms from the contralateral ear. It has been suggested that a low-amplitude pulse-synchronous waveform may be physiologic (10). Third, and similar to the last point, we would not have expected patients with bruxism or an intracanalicular vestibular schwannoma to have a pulse-synchronous pattern. Thus, there may be additional pathology unidentified, or findings may simply be physiologic. Given the lack of a rigid systematic protocol in the evaluation of PT with a pulse-synchronous APT waveform and the inability to further analyze differences in waveform amplitude for each pathology, future prospective studies are needed to better understand pathophysiologic mechanisms.

### Limitations

This single-center retrospective review is limited by several factors. First, with a comprehensive patient identification method, we likely captured patients within the cohort with only secondary or tertiary complaints of PT, which might have contributed to the low diagnostic rate and low APT screening utility values. Second, APT tracings were collected over a 20-second interval, a shorter time frame than previously reported, which may have led to false negatives. APT was performed by several audiologists, introducing potential for performance variations. Additionally, the decision to categorize APT tracings as pulse-synchronous if greater than half of the time a pulse-synchronous pattern was followed may have led us to incorrectly label patients as having negative tracings if their tracings displayed a pulse-synchronous pattern less than 50% of the time, leading to the high false negative rate and resulting in the low sensitivity and NPV observed. Fourth, objective PT (ie, that heard from a stethoscope) was not systematically recorded as a separate variable, but if systematically studied in the future, could modify the screening utility of APT waveforms. A final major limitation is the imperfection of radiologic studies and interpretations as gold diagnostic standards. External interpretations and imaging quality varied, limiting consistency across the cohort. These differences, combined with a lack of standardized imaging protocols, precluded reliable subset analysis of imaging findings. While blinded review of radiology data was considered a priori, we intentionally did not pursue this because it would not have mimicked the reality of practice and would have reduced generalizability.

## CONCLUSION

While APT is a simple, accessible, and noninvasive tool present in most otolaryngology clinics, APT had a low sensitivity and specificity for a radiologic correlate and surgeon diagnosis of subjective PT in this cohort. Clinicians should be encouraged to pursue imaging regardless of a negative APT tracing and may consider limiting further imaging studies if those done previously were unrevealing, even in patients with a pulse-synchronous waveform. This study encourages further investigation of APT as an effective screening tool across the full scope of pathologies associated with PT.

## Funding Sources

None declared.

## Conflicts of Interest

None declared.
